# Building capacities in the Andean region: Training health professionals on climate change and health

**DOI:** 10.1016/j.joclim.2025.100551

**Published:** 2025-08-22

**Authors:** Gilma Mantilla, Bertha Pineda, Cecilia Sorensen, Haley Campbell, Nicola Hamacher, Karen Glatfelter

**Affiliations:** a Global Consortium on Climate and Health Education, Department of Environmental Health Sciences, Mailman School of Public Health, Columbia University, NY, United States; b Organismo Andino de Salud, Convenio Hipólito Unanue, Lima, , Perú; c Lawrence General Hospital, MA, United States

**Keywords:** Capacity building, Climate and health education, Health professionals, Andean region, Training, Spanish-speaking

## Abstract

**Introduction:**

As the impacts of climate change increase, health professionals must understand its impact on disease and public health risks. Building capacity across various regions is essential for enhancing decision-making within the health sector and mitigating climate-related risks. In response, the Organismo Andino de Salud (ORAS-CONHU), the Global Consortium on Climate and Health Education (GCCHE) and the Panamerican Health Organization (PAHO) collaborated to launch the first virtual Andean regional course on Climate and Health.

**Case Presentation:**

This five-week course featured twice-weekly 90-minute live virtual sessions, followed by Q&A. Participants were administered pre- and post-surveys to evaluate the course's effectiveness. Out of 950 registered individuals, 767 attended at least one session, and 418 attended six or more sessions.

Results indicate increased capacity among health professionals to address climate resilience, including skills in vulnerability assessment, risk management, adaptation strategies**,** and effective communication.

**Discussion:**

This program is the first Spanish-language live virtual training targeting regional health officials from the Ministries of Health, National Institutes of Health, and international agencies involved in health promotion, disease surveillance and control, disaster management, health service provision, public health program management, and the evaluation of climate-sensitive diseases. While other institutions offer climate and health courses, these programs are primarily conducted in English and are mostly not tailored to regional challenges.

**Conclusion:**

The strong turnout of participants underscores a significant interest in this educational format and highlights the need for broader engagement in climate and health education.

## 1 Introduction

Climate change significantly impacts global health, requiring health professionals to understand and analyze its risk to disease and public health. These impacts vary by location, hazard (floods, droughts, storms, heat waves, wildfires), exposure levels, and existing vulnerabilities like socioeconomic status and healthcare access. These factors collectively determine community impacts and health system functioning. Enhancing capacity in various regions will strengthen health sector decision-making, mitigate climate-related risks, and improve healthcare systems in a changing climate [1,2].

As trusted voices in society, health professionals must be knowledgeable about climate issues to respond with early detection and investigation [3] to protect individual and community health against increasingly costly, complex emerging risks. Surveys of health professionals from all backgrounds conducted at global, regional, and national levels have revealed significant gaps in knowledge, educational programs and curricula [4]. Institutional knowledge of health protection measures is limited for various reasons, including the absence of structured climate change and environmental health content in health professional degree programs, as well as insufficient funding for research, particularly in low-income countries [[5], [6], [7], [8], [9]].

Health ministries, national health institutes, and academic communities urgently need to improve understanding of climate impacts on health and develop cross-sector adaptation strategies. This includes integrating climate and health data for vulnerability analyses and implementing interventions like early warning systems [10]. A key component of climate variability and change adaptation is training professionals to understand the role of climate in driving disease burden and impacting economic growth. In recent years, interest in incorporating climate change into health and public health education has significantly increased, often driven by students and universities [[11], [12], [13], [14], [15], [16], [17], [18], [19], [20], [21], [22], [23]].

In response, the Organismo Andino de Salud, the Andean Health Committee for Disaster Risk Management and Climate Change, the Global Consortium for Climate and Health Education, and the Subregional Program for South America of the Pan American Health Organization developed the Andean Climate and Health Course as part of the human resource capacity-building strategy outlined in the Andean Health and Climate Change Plan 2020–2025.

## 2 Case presentation

The free, five-week online certificate course (August 30-September 20, 2023) was designed for health professionals from the Ministries of Health, National Institutes of Health, and international cooperating agencies involved in health promotion, disease surveillance and control, disaster management, health service provision, public health program management, and the evaluation of climate-sensitive diseases. Registrations were submitted online, and networks of affiliated organizations were utilized to disseminate the course announcement.

Learning Objectives included: 1. Understand the relationship between health and climate change. 2. Recognize direct and indirect climate impacts on health. 3. Learn methodologies linking climate to health impacts, including data utilization, vulnerability analyses, and surveillance systems. 4. Exchange experiences and best practices. The format featured twice-weekly 90-minute live virtual sessions with theoretical content, case studies focused on Andean countries, and Q&A sections. Expert lecturers were selected for their specialized knowledge. Supplementary materials and session recordings were provided for asynchronous learning.

Key topics covered included: climate change and health, climate threats and their health impacts, tools for managing and integrating climate and health data, methodologies for climate risk assessment in health, adaptation and mitigation strategies, and communication. (Table 1)

**Table 1:** Curriculum outline of the Andean Regional course on Climate and Health.

Session	Topic
1	Climate change and health outlook
2	Tools for the management and integration of climate data in health Case study: Forecasts of El Niño and La Niña in the Andean countries (intersectoral view).
3	Methodology for assessing climate risk in health: Vulnerability and adaptation Case Study: Climate Resilience in Duran. Ecuador
4	Tools for vulnerability and risk analysis Case Study: Use of Data Library to prepare vulnerability and risk maps in climate sensitive events in the Andean region
5	Communicable diseases and Climate Change Case Study: Use of the weather forecast in the Climate and Health Bulletin. Colombia.
6	Non-communicable Diseases and Climate Change Case Study: Early warning system for extreme temperatures. Argentina
7	Mental Health, Migration and Climate Change Case Study: Advisory Opinion of the Republic of Chile and the Republic of Colombia on Climate Emergency
8	Adaptation, Climate Mitigation and Co-benefits. Case Study: Roadmap for a National Climate and Health Observatory. Argentina
9	Adaptation strategies to climate change from different levels of management in the health sector. Case Study: Adaptation instruments and measures for the province of Neuquén, Argentina
10	Intersectoral mitigation actions Case Study: Carbon footprint monitoring and reporting tools in the Hospital Sector

Source: Prepared by authors. All contents are available at: https://www.publichealth.columbia.edu/research/programs/global-consortium-climate-health-education/courses/past-courses/curso-andino-de-clima-y-salud.

Evaluation occurred through multiple methods: a pre-course survey that established baseline knowledge, a final exam that assessed learning gains, a post-course survey, and a 300-word reflection where participants applied course content to their work. Feedback surveys evaluated content relevance and overall satisfaction.

Participants who attended 6 or more sessions, passed the final exam (achieving a score of at least 70 %), and submitted a reflection on the applicability of the course content in their work were awarded a certificate of participation.

### 2.1 Results

Of 950 individuals registering, 767 participated in at least one and 418 attended six or more sessions with 282 individuals noting this was their first experience regarding this educational material. Peru and Ecuador had the highest participation rates, with 27.5 % and 20.5 %, respectively, followed by Chile (16.3 %), Venezuela (16.1 %), Colombia (11.5 %), Bolivia (6.5 %), and other countries (1.6 %). Most participants were from the government/public health sector (66.5 %), followed by government/other sectors (28 %) and international cooperation organizations (5.5. %). Table 2 details job focus of participants. The course was primarily advertised through the members of the Andean Committee of Emergency and Disasters and may have unintentionally drawn individuals inclined toward, environmental health or climate change and health-related initiatives and education.

**Table 2:** Participant performance area.

Area	N ( %)
Environmental Health	101 (24.2 %)
Emergencies and Disasters	67 (16.0 %)
Promotion and Prevention	51 (12.2 %)
Provision of Health Services	40 (9.6 %)
Public Health Surveillance	39 (9.3 %)
Other	38 (9.1 %0
Public policies	32 (7.7 %)
Research	24 (5.7 %)
Planning	10 (2.4 %)
Clinical Laboratory	7 (1.7 %)
Information and Technology	4 (1.0 %)
Reproductive Health	2 (0.5 %)
Finance	1 (0.2 %)
Human Resources	1 (0.2 %)
Child Health	1 (0.2 %)

#### 2.1.1 Final exam and Pre- and post-course surveys

A total of 362 participants took the exam, with an 87 % pass rate. However, of those who passed the exam, only 279 (77 %) had attended six or more sessions. Table 3 details results to survey data from participants who attended 6 or more sessions during the course and passed the final exam.

**Table 3:** Pre- and Post course surveys.

Q1: How often do you contemplate talking to your colleagues/patients/community about climate change and health?	At the time of registration	At the end of the course	Change
	(n) %	(n) %	(n) %
Frequently	(161) 57.7 %	(242) 86.7 %	(+81) 29 %
Sometimes	(83) 29.7 %	(30) 10.8 %	(−53) 18.9 %
Rarely	(33) 11.8 %	(2) 0.7 %	(−31) 11.1 %
Never	(2) 0.7 %	(5) 1.8 %	(+3) −1.1 %
Q2: How often will you use climate change and health knowledge and skills in your work?	(n) %	(n) %	(n) %
Frequently	(140) 50.2 %	(235) 84.2 %	(+95) 34 %
Sometimes	(98) 35.1 %	(39) 14.0 %	(−59)- 21.1 %
Rarely	(35) 12.5 %	(4) 1.4 %	(−31) −11.1 %
Never	(6) 2.2 %	(1) 0.4 %	(−5) −1.8 %
Q3: How confident do you feel about leading a project related to health and climate change in your institution/practice/community?	(n) %	(n) %	(n) %
Confident	(140) 50.2 %	(155) 55.6 %	(+15) +5.4 %
Somewhat Confident	(113) 40.5 %	(107) 38.4 %	(−59) −2.1 %
Not very confident	(24) 8.6 %	(16) 5.7 %	(−8) −2.9 %
Definitely no confident	(2) 0.7 %	(1) 0.4 %	(−1) −0.3 %

Regarding the likelihood of discussing climate change and health topics with colleagues, participants reported feeling more confident discussing the topic of climate change and health after taking the course, with an increase of nearly 29 % in the "frequently" category.

From the second survey question, it is clear that the number of individuals who plan to use their skills and knowledge frequently increased, while the other categories saw a decrease in numbers.

In the third question, the "confident" category percentage increased slightly while the “somewhat confident” and “not very confident” categories decreased slightly compared to the beginning of the course. This may be due to participants gaining a more precise understanding of the subject, leading to less biased responses than at the start.

#### 2.1.2 Reflections

Out of the total participants, 351 submitted reflections. Below are some examples of reflections from professionals across various fields in the Andean countries:

1. “The Andean Course on Climate and Health (ACCH) has strengthened my knowledge and experience in climate change, enhancing my understanding of conceptual and technical components, appropriate terminology, and my professional perspectives. I plan to apply this knowledge in developing technical regulations in Bolivia related to the National Health and Climate Change Plan and adaptation projects in the health sector, involving various decentralized units of the Ministry of Health and Sports.” [Environmental Health and Climate Change Risk Technical Professional, Ministry of Health and Sports, Bolivia]
2. “The ACCH was incredibly enriching, as it helped me understand the relationship between climate change and health. It strengthened my methodological and conceptual skills, particularly in risk assessment through threat, exposure, and vulnerability, as well as in managing climate data and analyzing health conditions related to communicable and non-communicable diseases. I also learned about developing early warning systems for phenomena like El Niño and their potential health implications, the effects of climate change on vector-borne diseases, and the importance of community engagement in health initiatives. I plan to utilize this knowledge to enhance our local health programs and advocate for policies that address the health impacts of climate change in my region.” [Public Health Officer, Ministry of Health, Peru].
3. **“**This course has been a transformative experience for me. It has not only deepened my understanding of the intricate links between climate change and health but also equipped me with practical tools to address these challenges. I intend to implement community-based projects that focus on educating the public about the health risks associated with climate change, particularly in rural areas where access to information is limited.” [Community Health Worker, Ecuador]

#### 2.1.3 Evaluation of course content

Of participants, 274 (98.2 %) felt that the proposed learning objectives were met; 273 (97.8 %) indicated that the support materials aided in understanding the course content; and 269 (96.4 %) found the reflective exercises useful for grasping the material. When rating the course on a scale of 1 to 5, where 5 was the highest, 211 (75.5 %) rated it a 5, while 64 (22.9 %) rated it a 4, and only 4 (1.4 %) rated it as 3. In response to the open-ended questions regarding the topics they liked most, the vast majority of participants noted all topics were relevant and particularly appreciated the inclusion of case studies with each subject.

## 3 Discussion

Health professionals are on the front lines of climate change and environmental hazards, but lack of education and training are barriers for them to engage and adapt to these realities [24,25]. The ACCH successfully addressed this gap by providing 418 health professionals from six Andean countries with essential knowledge and tools, with 279 completing all requirements. Key areas of interest included vulnerability assessment, heat wave warning systems, risk assessment tools, mental health impacts, and communication strategies. The reflections demonstrate a commitment to integrating climate health considerations into various sectors, ultimately contributing to improved health outcomes and resilience against climate impacts.

Given the urgent challenges posed by climate change, courses like this, using evidence-based guidelines [26], exemplify the importance of partnerships in building capacity and promoting climate resilience. A study evaluating three large-scale online courses on climate and health has demonstrated the efficacy of this approach in facilitating capacity-building and promoting equitable access to quality education on a global scale. A diverse international audience, including numerous participants from low-income countries, underscores the potential of these courses to disseminate quality educational content worldwide [27].

To our knowledge, this is the first Spanish language live virtual course explicitly designed for the Andean region to prepare health professionals from the Ministries of Health, National Institutes of Health, and international cooperating agencies involved in relevant sectors with the necessary knowledge, tools, and methodologies to address climate-sensitive health issues. As such, it is difficult to make direct comparisons with other studies. Institutions such as Imperial College London, Yale University, WHO Academy, and Harvard University offer publicly accessible climate and health-related courses online; however, programs are typically only available in English and are not tailored to the specific characteristics and challenges of different regions, except for the Yale course, which has some region-specific modules [[28], [29], [30], [31]].

Pre and post-survey findings generally align with the results of similar region-based climate and health education and capacity-building courses designed and organized by GCCHE [20]. In the present regional course, longitudinal changes showed an increase in confidence and intention to communicate, engage, and apply learned concepts. The course significantly contributed to "Strengthening the Capacities of Human Resources" as outlined in the Andean Plan for Health and Climate Change, while advancing cooperation networks and integration among Andean countries [32].

### 3.1 Limitations and areas for improvement

Limitations of the course included the self-selection of participants and incomplete participation. This restricts our ability to fully understand the changes in participants' confidence and their intentions to communicate, engage, and apply the concepts learned.

Recommendations for course improvement included extending question-and-answer sessions, providing more opportunities for direct interaction, adjusting schedules to avoid conflicts with work commitments, and considering hybrid or in-person formats for future courses. Participants also suggested developing an additional course focused on time series analysis and proposal writing methodologies. It is essential to continue supporting these professionals to implement the knowledge gained and foster collaboration among sectors to address the multifaceted challenges posed by climate change on health

## 4 Conclusion

The ACCH equiped participants with the knowledge and skills needed to address the intersection of climate change and public health. There was a strong interest in this education which highlights the potential to engage a broad audience of professionals through online education. There is a global deficiency in climate change and health education, with even fewer opportunities available in the Global South. This course serves as a significant step toward reducing educational inequity.

By implementing the recommendations outlined above, future courses can build on this success, further enhancing the learning experience and fostering a community of practice among health professionals dedicated to addressing climate-related health issues. Participants’ enthusiasm for the course and their commitment to applying what they learned in their respective fields underscore the importance of continued education in this critical area. Climate change will continue to significantly impact the health sector and live-virtual, competency-based, and regionally specific courses have the potential to contribute to positive change.

## CRediT authorship contribution statement

**Gilma Mantilla:** Writing – original draft, Supervision, Project administration, Methodology, Investigation, Formal analysis, Conceptualization. **Bertha Pineda:** Writing – review & editing, Methodology, Conceptualization. **Cecilia Sorensen:** Writing – review & editing, Investigation. **Haley Campbell:** Writing – review & editing. **Nicola Hamacher:** Formal analysis, Data curation. **Karen Glatfelter:** Writing – review & editing, Methodology.

## Declaration of competing interest

The authors declare that they have no known competing financial interests or personal relationships that could have appeared to influence the work reported in this paper.

Gilma Mantilla and Cecilia Sorensen are Editorial Board Members for The Journal of Climate Change and Health and were not involved in the editorial review or the decision to publish this article.
